# Effectiveness of Remote Family Education and Support Program for Parents of Adolescents With Eating Disorders Based on Interpersonal Psychotherapy: A Randomized Controlled Trial

**DOI:** 10.1002/eat.24541

**Published:** 2025-09-03

**Authors:** Fujika Katsuki, Hanayo Sawada, Yuka Kawasaki, Masaki Kondo, Atsurou Yamada, Norio Watanabe

**Affiliations:** ^1^ Nagoya City University Graduate School of Nursing Nagoya Japan; ^2^ Nagoya City University Hospital Nagoya Japan; ^3^ Japan Depression Center Tokyo Japan; ^4^ Nagoya City University Graduate School of Medical Sciences Nagoya Japan; ^5^ Soseikai General Hospital Kyoto Japan

**Keywords:** active listening, adolescents, eating disorders, interpersonal psychotherapy, interpersonal roles, parents, role transition

## Abstract

**Objective:**

Given the frequent conflict between parents and adolescents with eating disorders, we aimed to develop a remote family education and support program (rFESP) for parents based on the principles of interpersonal psychotherapy (IPT) and investigate its effect on promoting effective communication at home.

**Method:**

A clinical trial involving 67 parents of patients with adolescent eating disorders was conducted. Participants were randomly assigned to either the rFESP intervention group or the waiting control group. The intervention group received four rFESP sessions of 150 min each, conducted weekly over 4 weeks. Specifically, the primary outcome was the change in parents' active listening ability, as measured by the Active Listening Attitude Scale, while the secondary outcome was the change in the perception of social support and eating disorder symptoms evaluated by parents. Intention‐to‐treat analyses were conducted.

**Results:**

A statistically significant effect of rFESP was found on the Active Listening Attitude Scale at 8 weeks (mean difference: 3.68, 95% confidence interval: 1.89–5.48, *p* < 0.001). Similarly, the intervention group scored higher with regard to perceived social support (difference = 2.69, 95% confidence interval: 1.11–4.27, *p* < 0.001). No significant differences were found in eating disorder symptoms.

**Discussion:**

To the best of our knowledge, this is the first study to evaluate the effectiveness of an IPT‐based program for parents of adolescents with eating disorders. These findings suggest that this type of intervention is effective, albeit indirect, and could be a new support method for adolescent patients and their parents.

**Trial Registration:**

Clinical Trials.gov ID: NCT05840614

**Summary**

- Since conflict and confusion frequently arise between adolescents and their parents regarding adolescent eating disorders, we developed a remote family education and support program for parents based on the principles of interpersonal psychotherapy.
- After the program, there was a significant improvement in parents' active listening ability 4 weeks after the intervention.
- Parents also reported an increased perception of social support.

## 1 Introduction

Eating disorders are severe mental illnesses that emerge during adolescence and are associated with higher mortality rates than other mental disorders (van Eeden et al. 2021). Despite advances in treatments, mortality rates for anorexia nervosa (AN) and bulimia nervosa remain significantly high. Even after receiving inpatient treatment, individuals with AN still face more than a fivefold increased mortality risk (van Hoeken and Hoek 2020). Interpersonal problems, including avoidance, hostility, and intrusiveness, are common among patients with eating disorders (Troop et al. 2003). They are known to contribute to the persistence of eating disorder symptoms. Besides these, interpersonal distrust and avoidance can lead to interpersonal conflict, contributing to a vicious cycle where interpersonal problems grow and relationships deteriorate (Geller et al. 2018). Moreover, the degree of interpersonal problems is positively associated with more concerns over eating, shape, and weight, as well as the severity of bulimic behaviors (Hartmann et al. 2010). Interpersonal problems have been defined as a core component of AN and a risk factor for the development and maintenance of the disorder (Connan et al. 2007).

While the parental relationship is an important interpersonal relationship for adolescents with eating disorders, parents often find themselves entangled in prolonged interpersonal conflicts (Treasure et al. 2008; van Hoeken and Hoek 2020). The family members of patients with eating disorders, especially mothers, experience a heavy psychological burden and are at risk of poor mental health (Anastasiadou et al. 2014; Ohara et al. 2016). A comparison of caregivers of patients with eating disorders (44.2% of whom were mothers) with those caring for patients with depression or schizophrenia indicated that the former experienced a greater burden in terms of worry, tension, and pressure (Martín et al. 2015).

In a systematic review of the expressed emotion (EE) in eating disorders, caregivers were found to frequently exhibit high levels of EE, particularly emotional overinvolvement (EOI) (Anastasiadou et al. 2014). As an index reflecting the familial relationship, EE is assessed by examining the emotions expressed toward the patient by family members. The EOI of EE is evaluated based on self‐sacrifice, devotion, overprotection, and excessive displays of affection. Behaviors of parents of patients with eating disorders, characterized by excessive attention, overadaptation to the situation, or attempts to conceal negative patient outcomes that manifest as EOI, contribute to the worsening or maintenance of eating disorder symptoms (Kyriacou et al. 2008; Treasure et al. 2008). Patients with eating disorders cause anxiety in their parents by refusing to eat, leading parents to go to great lengths to encourage food intake. Moreover, patients' worsening symptoms also contribute to parental anxiety and overprotection. This vicious cycle may be related to two aspects. First, patients with eating disorders have been found to lack confidence in identifying their thoughts and feelings (Leppanen et al. 2022; Wollast et al. 2022) and may thus struggle to assert their thoughts and desires appropriately (Hartmann et al. 2010; Troop et al. 2003; Williams et al. 1993). Consequently, communication between patients and their parents may become conflicted and confused. Second, adolescents with eating disorders and their parents have difficulty with domestic communication (Erriu et al. 2020). Based on our clinical experience, both parents and adolescents may struggle to adjust to the role transition that accompanies adolescents' physical and mental growth.

Interpersonal psychotherapy (IPT) is a psychotherapy originally conceptualized for depression (Weissman et al. 2000). IPT for depression consists of four interpersonal therapeutic foci, including “interpersonal role disputes with different expectations” and “role transitions.” In addition, family‐based IPT (FB‐IPT) was developed as a treatment approach for preadolescent depression (Dietz 2020; Dietz et al. 2015). FB‐IPT focuses on improving parent–patient conflict to decrease preadolescent depression. Considering the characteristics of adolescents with eating disorders, their parents are likely to encounter issues related to interpersonal role disputes with different expectations and role transitions within the IPT therapeutic framework. To this end, we developed a family education and support program exclusively for the parents of adolescents with eating disorders using the IPT concept, focusing on interpersonal role disputes and differences in expectations and role transitions. Moreover, a family education and support program employing family psychoeducation, which is a method of working with families supporting persons with mental illness, has been associated with relapse prevention or symptom improvement (Bighelli et al. 2021; Katsuki et al. 2022; Lyman et al. 2014). For instance, studies on family psychoeducation for eating disorders have shown that caregivers report an increase in knowledge about eating disorders, an enhancement in their self‐efficacy, and a reduction in EE, particularly EOI, as well as psychological and family distress (Kurnik Mesarič et al. 2024; Uehara et al. 2001). Previous studies, including Supporting Carers of Children and Adolescents with Eating Disorders, have attempted to enhance parent–child communication, which may be effective (Franta et al. 2018; Philipp et al. 2020). However, no study has addressed the vicious cycle of communication within the home by directly correcting differences in role expectations to improve parent–child interactions.

To the best of our knowledge, this is the first study to examine the effectiveness of a remote family education and support program (rFESP) to promote effective communication within homes using a randomized controlled trial design, focusing on the active listening skills of parents of patients with adolescent eating disorders. In Japan, eating disorders, particularly restricting‐type AN, have increased in physical and psychological severity (Harada et al. 2021). The evidence‐based treatment system for eating disorders, such as family‐based treatment in Western countries, is inadequate (Iguchi et al. 2021), with only five designated treatment hospitals nationwide. Many patients cannot access appropriate care due to the limited availability of hospitals specializing in eating disorder treatment. Therefore, this program, which utilizes a remote system, is a practical solution. We hypothesized that parents receiving the rFESP would show greater improvements in active listening skills, social support, loneliness, mental health status, family functioning, and anorectic behavior.

## 2 Methods

### 2.1 Design Overview

The present study was conducted with the parents of patients with eating disorders, who were allocated to one of two groups: (1) rFESP in addition to treatment‐as‐usual for patients and (2) treatment‐as‐usual for patients only. Treatment‐as‐usual consisted of either consultation with a physician or no treatment. The primary endpoint was improvement in parents' active listening ability, as measured by the Active Listening Attitude Scale (ALAS) at 8 weeks.

This study was approved by the Ethics Review Committee of the Nagoya City University Graduate School of Nursing (ID: 22033‐4). All parent participants were requested to provide written informed consent after the study's purpose and procedures were explained. Informed consent was not obtained from the patients, as the study involved no direct intervention or evaluation of them. This study was registered at ClinicalTrials.gov under the number NCT05840614. The protocol for this trial was reported previously (Katsuki et al. 2024). This study adheres to the CONSORT guidelines (Schulz et al. 2010).

Participants were recruited using a website and flyers and signed up via email. Eligible participants were provided with an ID number and asked to provide informed consent and complete a baseline assessment. After the assessment, participants were randomized. Assessments were performed at baseline (Assessment I), before randomization, and at 4 weeks (Assessment II), and 8 weeks (Assessment III) after randomization. The trial period was from Weeks 0 to 4, and the follow‐up period was from Weeks 4 to 8. Assessment II was performed at the Week 4 evaluation date ±5 days, and Assessment III at the Week 8 evaluation date ±5 days.

### 2.2 Eligibility Criteria

The target population was parents of patients with adolescent eating disorders. Parents may or may not have been biological parents. The inclusion criteria were (1) parents of patients diagnosed with eating disorders (AN and BN) by a physician, (2) parent‐rated Anorectic Behavior Observation Scale (ABOS) score > 8 points at enrollment, (3) patient age between 12 and 29 years at enrollment, and (4) parents who live with patients at the time of enrollment and are expected to live with them during the study period. Parents and patients may or may not have had other psychiatric comorbidities. The exclusion criteria were (1) parents who cannot read or write in Japanese, (2) parents who cannot use the Zoom online meeting system, and (3) study investigators and their families. Patients may or may not have received treatment. This decision was based on the desire to adopt the study results to be effective for families with eating disorders at all stages of treatment.

### 2.3 Randomization

The participants were randomly allocated to one of the two groups at a 1:1 ratio. A computer randomization system generated random allocation sequences using minimization and stratified participants according to their ability to engage in active listening (ALAS score cutoff of 36 points).

### 2.4 Treatment

#### 2.4.1 Intervention

The rFESP therapy was divided into four sessions (Table 1). Each session consisted of a lecture, followed by role‐playing and supportive group therapy for a duration of approximately 150 min. The groups comprised approximately six to nine members and met online once a week over the course of 4 weeks. The rFESP consisted of IPT elements and family psychoeducation, which provides information as well as developing providing information. It also focuses on the development of problem‐solving, communication, and coping skills. Participants were provided information on symptoms of eating disorders with the goal of externalizing the illness in each session. In particular, the third and fourth sessions explained the mechanism of interpersonal role disputes with differences in expectations and the role transition with adolescent growth using an adolescent eating disorder case. Subsequently, participants role‐played using IPT communication skills. Supportive group therapy used problem‐solving, focusing on members' strengths and coping ideas. After the last session, a chat group was created, whereby participants could maintain their own self‐help support. Participants assigned to the waiting control group did not receive any intervention.

**TABLE 1:** Overview of remote family education and support program content.

	Lecture	Role‐play	Support group therapy
	30 min	30 min	90 min
Session 1	Information on the symptoms of eating disorders and the mechanism of IPT	None	Supportive group therapy using problem‐solving focuses on members' strengths and coping ideas Families were socializing with other families to warm up Group members presented their own problems or goals For each problem or goal, group members discussed and suggested possible solutions based on the person's strengths
Session 2	Information on the characteristics of adolescents, focusing on adolescent role transitions	None	
Session 3	Information on effective communication according to IPT We explained the mechanism of interpersonal role disputes with differences in expectations using a case	Role‐play using IPT communication skills Group members imagined how the parent in the case feels in one scene of an eating disorder parent–patient case and shared it with group members In the same scene, group members imagined what the parent in the case is expecting from the patient and shared it with group members In the same scene, group members imagined how the patient in the case feels and shared it with group members In the same scene, group members imagined what the patient in the case is expecting from the parent and shared it with group members In the same scene, group members thought about what they would say to this patient and shared it with group members Role play: one of the group members played the role of the parent	
Session 4	Information on effective communication according to IPT We explained the mechanism of role transition due to adolescent growth using a case	Role‐play using IPT communication skills Above, (i)–(iv) are the same v In the same scene, group members thought about what they would say to this patient, being aware of the role transition that accompanies adolescence, and shared it with group members iv Role play: one of the group members played the role of the parent	

Abbreviation: IPT, interpersonal psychotherapy.

#### 2.4.2 Therapist Training/Supervision and Fidelity Control

Four therapists conducted the program, all of whom were registered nurses. The first author (F.K.) is a nurse and family therapist certified by the Japan Network of Psychoeducation and Family Support Program (JNPF), with an IPT first‐class workshop certification. The second author (H.S.) is a nurse and psychologist. The third author (Y.K.) is a certified nurse specialist in psychiatric‐mental health nursing. All four therapists have psychiatric nursing experience. The fourth author (M.K.) is a certified supervisor authorized by the International Society of Interpersonal Psychotherapy and supervised the program. All sessions were recorded on a computer to ensure fidelity, and 25% of each condition was randomly selected and evaluated by independent researchers.

### 2.5 Outcome Measures for Parents

#### 2.5.1 Baseline Characteristics

The following baseline sociodemographic characteristics were obtained, as shown in Table 2: (1) age (parents and patients), (2) patient's eating disorder duration, (3) patient's gender, (4) family relationship (father, mother, and other), and (5) whether parents participate in a family self‐help group.

**TABLE 2:** Characteristics of participants at baseline.

Characteristics	Intervention group, *n* = 34	Control group, *n* = 33	All participants, *n* = 67
Participants			
Age, mean (SD), years	49.7 (4.1)	50.7 (4.7)	50.2 (4.5)
Family relationship, *n* (%)			
Father	2 (5.8)	1 (3.0)	3 (4.5)
Mother	32 (94.1)	32 (96.9)	64 (95.5)
Parents' experience with joining a family self‐help group, *n* (%)			
Yes	24 (70.5)	20 (60.6)	44 (65.7)
Yes, in the past	4 (11.7)	5 (15.1)	9 (13.4)
No	6 (17.6)	8 (24.2)	14 (20.9)
ALAS, mean (SD)	33.1 (7.6)	33.9 (6.0)	33.5 (6.9)
ABOS, mean (SD)	28.4 (8.8)	24.6 (7.6)	26.5 (8.6)
SPS‐10, mean (SD)	30.7 (5.5)	30.0 (5.0)	30.4 (5.3)
ULS, mean (SD)	38.4 (9.3)	41.6 (9.1)	40.0 (9.4)
K6, mean (SD)	9.4 (5.2)	11.3 (4.9)	10.3 (5.1)
FAD, mean (SD)	28.4 (6.2)	27.3 (6.0)	27.8 (6.1)
Patient			
Age, mean (SD), years	17.4 (3.3)	18.5 (5.0)	18.0 (4.3)
Sex, *n* (%)			
Female	32 (94.1)	33 (100)	65 (97.0)
Male	2 (5.8)	0 (0)	2 (3.0)
Eating disorder duration, mean (SD), years	2.7 (2.2)	3.7 (3.5)	3.2 (3.0)

Abbreviations: ABOS, Anorectic Behavior Observation Scale; ALAS, Active Listening Attitude Scale; FAD; Family Assessment Device; K6, Kessler Psychological Distress Scale; SPS‐10, The Social Provision Scale‐10 item; ULS, University of California, Los Angeles Loneliness Scale.

#### 2.5.2 Primary Outcome Measure for Parents

Given that this program aimed to improve the confusion and stagnation in patient–parent communication within the home by focusing on enhancing parents' active listening abilities, parents' ALAS score at 8 weeks was selected as the primary outcome.

#### 2.5.3 Alas

The ALAS, developed by Mishima et al. (2000), was used to assess the listening attitude. The ALAS comprises 20 items on two subscales: listening attitude (10 items) and listening skills (10 items). Each item was scored from 0 to 3; the higher the score, the better the listening attitude or skill. Cronbach's *α* of the ALAS in a previous study was 0.855 (Yamada et al. 2021).

### 2.6 Secondary Outcome Measures for Parents

#### 2.6.1 ABOS

The ABOS is a questionnaire used to evaluate patients' eating behaviors based on information provided by their relatives. Many patients with eating disorders tend to deny their conditions and frequently drop out of treatment programs at clinics. In such cases, clinicians rely on information provided by their parents or relatives to assess the patient's condition. The ABOS, which was developed to evaluate a patient's symptoms based on the relatives' description of their eating behavior, is useful in clinical settings for evaluating patients with eating disorders. The ABOS consists of 30 items to be answered “yes” (2 points) or “no” (0 points) if relatives are certain of the information, or “?” (1 point) if relatives are uncertain. The higher the total score, the more pathological the subject's behavior is considered to be. The ABOS has three domains: (a) eating behavior, concern with weight and food, denial of the problem; (b) bulimic‐like behavior; and (c) hyperactivity. ABOS has a sensitivity of 90.0% and a specificity of 89.6% (Vandereycken 1992). The reliability and validity of the Japanese version of the ABOS have been confirmed with Cronbach's *α* value of 0.82 (Uehara et al. 2002).

#### 2.6.2 Social Provisions Scale‐10

The perception of social support was evaluated using the shortened version of the Social Provisions Scale (SPS), originally created by Cutrona and Russell (1987) and later shortened (SPS‐10) by Iapichino et al. (2016). A Japanese version of the SPS‐10 was also created (Katsuki et al. 2020). The SPS‐10 consists of 10 items and retains five of the six original SPS subscales: attachment (emotional support), social integration, reassurance of worth, reliable alliance (material support), and guidance. The total SPS‐10 score ranges from 10 to 40, with a higher score indicating a stronger perceived provision of social support. Cronbach's *α* was 0.809 for the original Italian version (Iapichino et al. 2016) and 0.89 for the Japanese version (Katsuki et al. 2020).

#### 2.6.3 University of California, Los Angeles Loneliness Scale

To assess loneliness, we used the Japanese version (Moroi 1992) of the University of California, Los Angeles Loneliness Scale (ULS), originally developed by Russell (1996). The ULS is a 20‐item scale, with each item scored from 1 to 4; the higher the score, the stronger the loneliness. Cronbach's *α* of the Japanese version was 0.885 (Moroi 1985).

#### 2.6.4 Kessler Psychological Distress Scale

To assess psychological distress, we used the Japanese version (Furukawa et al. 2008) of the Kessler Psychological Distress Scale (K6), originally developed by Kessler et al. (2003). The K6 is a 6‐item self‐reported scale developed to screen for depression and anxiety disorders based on definitions from the Diagnostic and Statistical Manual of Mental Disorders (Fourth Edition), and analyzes patients' symptoms over the last 30 days. Moreover, it can be used to quantify nonspecific psychological distress (Kessler et al. 2003). Items are rated from 0 to 4, with the total score ranging from 0 to 24; the higher the score, the more severe the psychological distress. The validity of the K6 has been analyzed and confirmed by two independent studies (Furukawa et al. 2003; Kessler et al. 2003), and the validity of the Japanese version has also been confirmed (Furukawa et al. 2008). Cronbach's *α* of the original tool was 0.89 (Kessler et al. 2003).

#### 2.6.5 Family Assessment Device

To assess family functioning, we used the Japanese version of the Family Assessment Device (FAD) (Saeki et al. 1997), developed by Epstein et al. (1983). While the FAD comprises seven subscales, this study only used the General Functioning subscale. Each item is scored from 1 to 4; the higher the score, the poorer the family functioning. Cronbach's *α* of the General Functioning subscale was 0.92 in the original study (Epstein et al. 1983) and 0.85 in another study (Hausken et al. 2019).

### 2.7 Sample Size and Statistical Power

The sample size was calculated based on the power analysis of the ALAS score. Effect sizes were estimated based on our previous cohort study (Katsuki et al. 2023). The change in ALAS scores from pretreatment to posttreatment (8 weeks after randomization) was 7.0 ± 6.0 (mean ± SD) in the intervention group, and 1 ± 6.0 in the waiting control group. With a power of 0.9 to detect a significant difference at *p* = 0.05 (two‐sided), it was calculated that 28 patients would be required for each arm. Thus, allowing for a 20% drop‐out rate, 35 participants were recruited per group.

### 2.8 Data Analysis Plan

The initial descriptive statistics summarized the participants' characteristics as means, standard deviations, minima, maxima, frequencies, and percentages. All the analyses were based on an intention‐to‐treat model. A linear mixed model with the baseline value of each variable as a covariate was used to examine the interactions (group*visit) and between‐group differences at each visit. The estimated means and 95% confidence intervals (CIs) for each group and between‐group differences at each visit were calculated. *p* values less than or equal to 0.05 were considered significant. All statistical analyses were performed using SPSS version 29.0.2.0 for Windows (IBM Corp., Armonk, NY, USA).

## 3 Results

### 3.1 Participant and Baseline Characteristics

**FIGURE 1:**
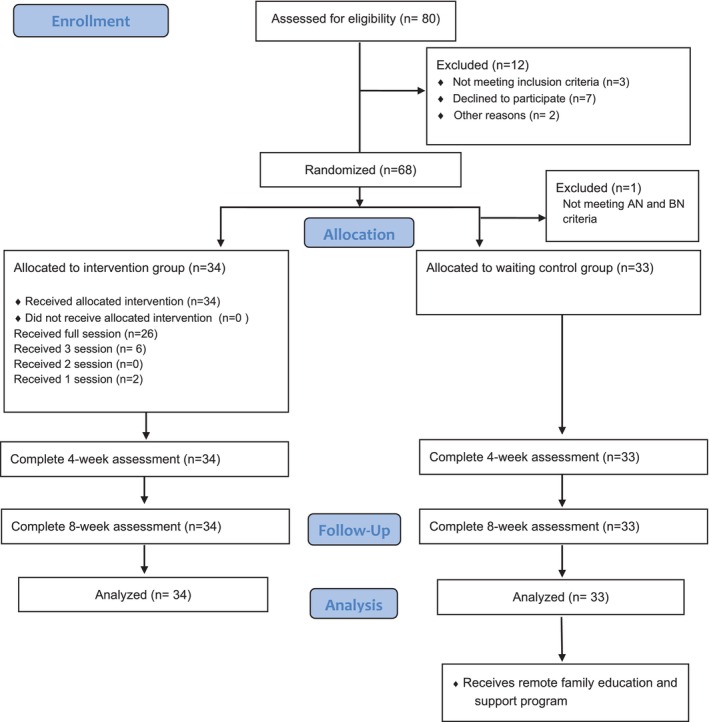
Participant flow diagram.

Of the 80 screened participants, 68 were randomized for this study. Twelve participants were excluded before randomization, among whom three had ABOS scores of ≤ 8, seven refused to participate, and two were unreachable. After randomization, one participant was excluded from the control group because the AN or BN criteria were not met. The 67 participants were then divided into the rFESP group (34 participants) and a waiting control group (33 participants). All participants were available for the analysis (Figure 1). Table 2 summarizes the sociodemographic and clinical data of the 67 participants at baseline. Of these, 26 participants attended the full program, while six participants attended three sessions. Of the four randomized sessions assessed for adherence in the intervention group, 100% of the quality checkpoints were met by the therapist.

### 3.2 Primary Outcome

**FIGURE 2:**
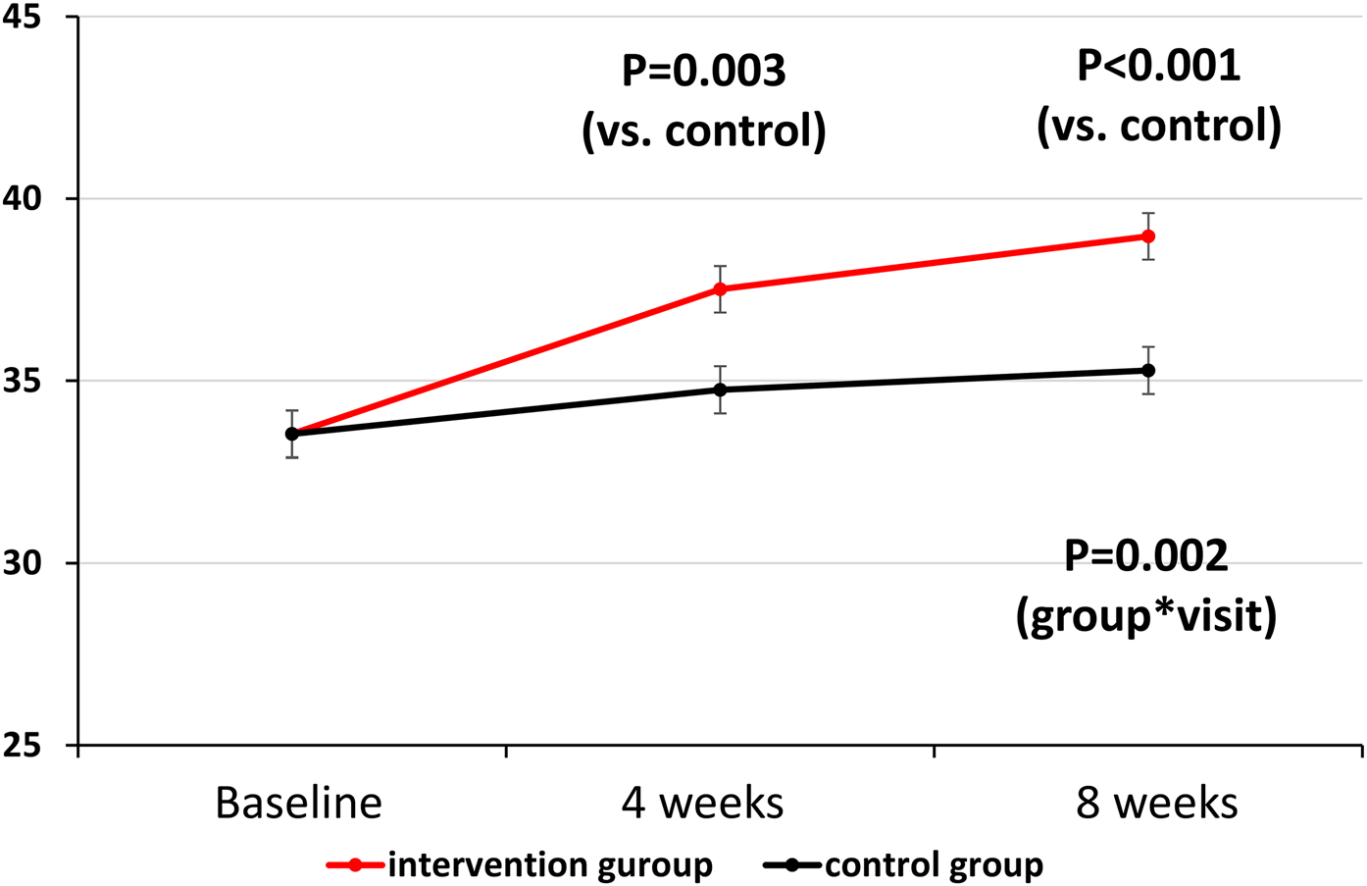
ALAS scores at baseline, 4 weeks, and 8 weeks.

Table 3 shows the participants' latest square (LS) means and their 95% CIs for the ALAS scores at 4 and 8 weeks. With respect to the primary outcome of the ALAS score at 8 weeks, there was a significant difference between the intervention and control groups (difference = 3.68, 95% CI: 1.89–5.48, *p* < 0.001; Figure 2).

**TABLE 3:** Estimated mean outcome scores at 4 and 8 weeks.

	Intervention group	Control group	Mean difference (intervention—control) [95% CI]	*p* (group*visit)	*p* (between‐group)
Questionnaire, LS mean [95% CI]					
ALAS				0.002	
4W	37.51 [36.25, 38.77]	34.75 [33.47, 36.03]	2.76 [0.96, 4.56]		0.003
8W	38.96 [37.70, 40.22]	35.28 [34.00, 36.56]	3.68 [1.89, 5.48]		< 0.001
ABOS				0.841	
4W	25.02 [23.58, 26.46]	24.25 [22.79, 25.71]	0.77 [−1.30, 2.84]		0.465
8W	23.02 [21.58, 24.46]	22.65 [21.19, 24.11]	0.37 [−1.70, 2.45]		0.722
SPS‐10				0.016	
4W	32.26 [31.13, 33.39]	30.83 [29.71, 31.95]	1.43 [−0.16, 3.02]		0.078
8W	32.45 [31.33, 33.57]	29.76 [28.64, 30.88]	2.69 [1.11, 4.27]		< 0.001
ULS				0.069	
4W	38.61 [37.11, 40.11]	39.31 [37.79, 40.83]	−0.70 [−2.85, 1.45]		0.520
8W	36.75 [35.25, 38.25]	39.59 [38.07, 41.11]	−2.84 [−4.99, −0.69]		0.010
K6				0.740	
4W	8.69 [7.65, 9.74]	8.86 [7.83, 9.89]	−0.17 [−1.65, 1.31]		0.820
8W	7.26 [6.23, 8.29]	7.94 [6.91, 8.97]	−0.68 [−2.16, 0.79]		0.360
FAD				0.423	
4W	25.88 [24.71, 27.04]	27.03 [25.88, 28.19]	−1.16 [−2.80, 0.49]		0.167
8W	25.22 [24.06, 26.37]	26.30 [25.14, 27.45]	−1.08 [−2.71, 0.56]		0.194

*Note*: Linear mixed model with the baseline value of each variable as a covariate was used to examine interactions (group*visit) and between‐group differences at each visit.

Abbreviations: 4W, 4 weeks; 8W, 8 weeks; ABOS, Anorectic Behavior Observation Scale; ALAS, Active Listening Attitude Scale; FAD, Family Assessment Device; K6, Kessler Psychological Distress Scale; LS means, latest square means; SE, standard error; SPS‐10, The Social Provision Scale‐10 item; ULS, University of California, Los Angeles Loneliness Scale.

### 3.3 Secondary Outcomes

With respect to the SPS‐10 scores at 8 weeks, the intervention group was significantly higher than the control group (difference = 2.69, 95% CI: 1.11–4.27, *p* < 0.001). No significant differences were observed with respect to the ABOS, K6, ULS, and FAD (Table 3).

## 4 Discussion

The present study examined the effectiveness of the rFESP using the IPT concept in parents of patients with adolescent eating disorders. There was a significant benefit of this intervention on the primary outcome, that is, the active listening ability of parents, at 4 weeks after the intervention. Moreover, there was also a significant improvement in perceived social support. However, we found no effect of this intervention on loneliness, mental health status, family functioning, or patients' eating disorder behavior as observed by parents.

Possible reasons why the rFESP intervention improved parental active listening ability include the following: (1) by understanding the IPT concept and role‐playing accordingly, parents were able to imagine the feelings and expectations of their children; (2) parents became aware of adolescent growth and avoided over‐interference; (3) by externalizing the illness, parents were able to listen to their children's ideas without guilt; and (4) through focusing on parents' own strengths in the group session, they were also able to become aware of their children's strengths. Moreover, the present study saw a significant improvement in perceived social support in the intervention group. In a previous cohort study, the degree of improvement in active listening attitudes among parents of patients with eating disorders was significantly greater in the improved social support group than in the non‐improved social support group (Katsuki et al. 2023). It is thus predicted that parents' sense of support from those around them helped them listen to their children's thoughts without anxiety. Adolescent patients and their parents tend to experience conflicts and difficulties in domestic communication. In such situations, the IPT concept (including interpersonal role disputes with different expectations and role transitions) may fit well.

In the rFESP intervention, despite the improvement in the perceived social support of parents, there was no significant improvement in mental health status as evaluated by the K6. In a previous study, mothers who felt that they received higher levels of social support felt less depressed, as evaluated by the Beck Depression Inventory‐II, than those with lower perceived levels of social support (Yamada et al. 2021). However, the study found no significant difference in the K6 scores between groups (Yamada et al. 2021). As the K6 is a screening scale for depression and anxiety, it may not have adequately detected the degree of depression. Similarly, K6 may not have been sensitive enough to detect changes in participants in this study. Several studies have reported that parents of patients with eating disorders, especially mothers, experience elevated levels of depression and anxiety (Gonzalez et al. 2012; Stefanini et al. 2019; Treasure et al. 2001). As such, it is important to focus on improving the mental health of parents themselves; aside from the need for parents to maintain their own mental health, parental anxiety is also known to trigger overprotection toward patients. Children's depressive symptoms and parents' moods were reportedly improved in a study on the FB‐IPT for preadolescents with depression. Acknowledging the improvement in children's symptoms may be necessary to enhance parents' mental health. A study on the FB‐IPT for preadolescents with loss‐of‐control eating reported greater reductions in disordered eating attitudes. This FB‐IPT intervention consisted of 12 weekly, 45‐min sessions delivered to parent–child dyads. If the same intervention as that in the rFESP is provided to patients simultaneously, it may be effective in improving eating disorder symptoms. The improvement trend in loneliness evaluated by the ULS (difference = −2.84, 95% CI: −4.99 to −0.69, *p* = 0.01) in the intervention group may be due to the effects of the chat group conducted after the intervention. This finding suggests the importance of continuous support from self‐help groups for parents of patients with eating disorders. In the current study, there was no improvement in FAD, and the rFESP intervention did not have an impact on the family system as a whole.

This study has some limitations. First, patients could not participate in the program. According to the FB‐IPT, treatment is best conducted with both the patients and their parents, particularly in the case of adolescents. Second, patients' information, including treatment status, comorbid diagnoses, or body weight, was unavailable. Participants were randomly assigned to each group using a computerized randomization system that generated allocation sequences. Therefore, we could expect all confounders, including the contents of treatment‐as‐usual and any unmeasured important factors at baseline, to be almost equally distributed in these two groups. Although patients' outcomes were not included for the reasons outlined above, their treatment status remained an important factor influencing the results. Given that treatment resistance and dropout are common features of eating disorders (Campbell 2009; Halmi 2013), we anticipated that obtaining responses from patients would be challenging. This can be considered a limitation of the current study, as it underestimated the patients' willingness to recover and their autonomy. Third, the average age of the patients was 18 years, and many patients were in their 20s, even though they were considered adolescents. However, patients with eating disorders usually fail to complete typical adolescent developmental tasks and may retain an adolescent mindset. Here, we focused on parents and patients living together; therefore, family conflicts may persist even when patients are in their late 20s. In addition, since developmental researchers have reached a growing consensus that adulthood is not fully established until the mid‐20s (Ledford 2018; Office for National and Statistics 2019), we considered individuals in their 20s as adolescents in this study. Although these studies focused on early psychosis and involved young participants with mean ages > 18 years, they demonstrated significant improvement in patients' psychiatric symptoms by involving only their parents (Lenior et al. 2001; Zhang et al. 1994). Given this evidence, the intervention program in our study—focused solely on family members—may be considered to have the potential to improve patient outcomes, despite the mean patient age being 18.0 years. Fourth, because the follow‐up period was short, it remains uncertain whether the effects will be sustained in the long term. Future studies may employ improved methods to evaluate long‐term outcomes. Fifth, the ratings were evaluated by parents using self‐report measures; therefore, potential biases associated with self‐report ratings exist. Finally, this study was not open to those who could not use remote systems.

Despite its limitations, this study has several strengths, namely that it was the first attempt to use the IPT concept for parental education and support for eating disorders. Second, we created a program focused on role transition through changes in adolescence. Third, this is a remote participation program; therefore, participants can join from anywhere in Japan. This is important considering that many patients do not receive adequate treatment in the country, as hospitals with eating disorder specialists tend to be far away. Fourth, we assessed parents' status as well as the eating disorder symptoms of patients, as measured by the ABOS. Finally, statistical analyses were performed by a blinded statistician.

To conclude, as the first study to evaluate the effectiveness of an IPT‐based program for parents of adolescents with eating disorders, our findings suggest that this type of intervention is effective, albeit indirect, and could serve as a new support method for both parents and adolescent patients.

## Author Contributions

**Fujika Katsuki:** conceptualization, data curation, writing – original draft, methodology, investigation, project administration, funding acquisition, resources, visualization. **Hanayo Sawada:** conceptualization, investigation. **Yuka Kawasaki:** investigation. **Masaki Kondo:** conceptualization, methodology, supervision, writing – review and editing. **Atsurou Yamada:** conceptualization, methodology. **Norio Watanabe:** conceptualization, methodology, formal analysis, supervision, writing – review and editing, validation.

## Ethics Statement

This study was approved by the Ethics Review Committee of Nagoya City University Graduate School of Nursing, Japan (Ref: No 22033‐4). All participants were provided written informed consent to participate in the study.

## Conflicts of Interest

Masaki Kondo received speaker's fees from Takeda Pharmaceutical Co. Ltd. and Sumitomo Pharma Co. Ltd. and research funds from Olive Union Co. Ltd. Atsurou Yamada is an employee of a course endowed by Nagoya City and received medical fees from Gifu Hospital, instructor fees from Asamidori no Kai, a social welfare corporation, committee member fees from Snowm Co. Ltd., speaker's fees from Aichi Education and Sports Foundation, Viatris Pharmaceutical Co. Ltd., Takeda Pharmaceutical Co. Ltd., Nobel Pharma Co. Ltd., Mental Care Association Japan, and other fees from Nagoya City. Norio Watanabe has received royalties from Sogensha, Medical View, and Advantage Risk Management for writing. The other authors declare no conflicts of interest.

## Data Availability

The data that support the findings of this study are available from the corresponding author upon reasonable request.
